# Future scenarios of marine resources and ecosystem conditions in the Eastern Mediterranean under the impacts of fishing, alien species and sea warming

**DOI:** 10.1038/s41598-018-32666-x

**Published:** 2018-09-24

**Authors:** X. Corrales, M. Coll, E. Ofir, J. J. Heymans, J. Steenbeek, M. Goren, D. Edelist, G. Gal

**Affiliations:** 1 Kinneret Limnological Laboratory, Israel Oceanographic & Limnological Research, PO Box 447, Migdal, Israel; 2 Institut de Ciències del Mar (ICM-CSIC), Passeig Marítim de la Barceloneta, n° 37–49, 08003 Barcelona, Spain; 3 Ecopath International Initiative Research Association, Barcelona, Spain; 4 Scottish Association for Marine Science, Scottish Marine Institute, Oban, PA 371QA Scotland; 5 European Marine Board, Wandelaarkaai 7, Oostende, 8400 Belgium; 6 Department of Zoology and The Steinhardt Museum of Natural History, Tel Aviv University, Tel Aviv, 69978 Israel; 7 Leon Recanati Institute for Marine Studies, Charney School for Marine Sciences, Faculty of Natural Sciences, University of Haifa, Mont Carmel, Haifa, 31905 Israel

## Abstract

Using a temporal-dynamic calibrated Ecosim food web model, we assess the effects of future changes on marine resources and ecosystem conditions of the Israeli Mediterranean continental shelf. This region has been intensely invaded by Indo-Pacific species. The region is exposed to extreme environmental conditions, is subjected to high rates of climate change and has experienced intense fishing pressure. We test the impacts of a new set of fishing regulations currently being implemented, a continued increase in sea temperatures following IPCC projections, and a continued increase in alien species biomass. We first investigate the impacts of the stressors separately, and then we combine them to evaluate their cumulative effects. Our results show overall potential future benefits of fishing effort reductions, and detrimental impacts of increasing sea temperature and increasing biomass of alien species. Cumulative scenarios suggest that the beneficial effects of fisheries reduction may be dampened by the impact of increasing sea temperature and alien species when acting together. These results illustrate the importance of including stressors other than fisheries, such as climate change and biological invasions, in an ecosystem-based management approach. These results support the need for reducing local and regional stressors, such as fishing and biological invasions, in order to promote resilience to sea warming.

## Introduction

Marine ecosystems have been increasingly altered worldwide by a diversity of global, regional and local anthropogenic stressors. These stressors include climate change, biological invasions, overexploitation, pollution and habitat destruction and often co-occur in time and space and have cumulative effects^1,2^. Such ecosystem changes can have large consequences on species abundance and distributions, marine biodiversity, and ecosystem functioning and services^3–5^.

Despite increasing knowledge about the impacts of single stressors on marine populations, habitats and ecosystems, the cumulative effect of multiple stressors remains largely unknown^6,7^. In addition, marine populations, habitats, and their ecosystems are affected by environmental fluctuations^8,9^. Therefore, understanding how multiple human threats, marine organisms, and ecosystems interact and influence each other is an issue of pressing importance. To address this challenge, a shift towards a more comprehensive analysis and management of human activities is required, as emphasised by the ecosystem-based management (EBM) approach^10,11^.

The EBM approach has sparked great interest among the scientific community and new tools have been developed in recent decades. Within this context, ecosystem modelling approaches have increasingly been adopted as useful tools to study marine ecosystems as a whole and to forecast ecosystem dynamics and develop and test future scenarios for marine ecosystems^12–14^.

Ecosystem models and ecological forecasts face several obstacles linked to ecosystem characteristics and include high uncertainty^15,16^. Nevertheless, they have the potential to contribute significantly to achieving goals in marine conservation and management by offering guidance to decision-makers^17^. Their use in assessments, policy support, and decision-making can provide insights into how the ecosystem could respond to plausible future stressors, enabling the development of adaptive management strategies, and allowing for exploration of the implications of alternative management options^13,18,19^.

One of the most commonly used ecosystem modelling software is Ecopath with Ecosim (EwE), which has been widely applied to model aquatic food webs^20,21^. This approach has been used to hindcast and forecast future human impacts on aquatic food webs, such as fishing^22^, and increasingly other stressors like climate change^23^ and biological invasions^24^. EwE has been applied within the scope of evaluating cumulative impacts of human activities^25^. For example, Serpetti, *et al*.^26^ assessed the cumulative impact of sea warming and sustainable levels of fishing pressure in the West Coast of Scotland. In addition, Libralato, *et al*.^27^ developed temporal simulations to explore the effects of the arrival of invasive species, changes in primary production and sea warming in the Adriatic Sea.

The Mediterranean Sea is a semi-enclosed sea that is highly impacted by anthropogenic activities^1,2^. The Mediterranean is a global hotspot of alien species^2,28,29^, especially its eastern basin due to the opening and continuous enlargement of the Suez Canal^30,31^. Currently, 821 species are described as established alien species in the Mediterranean Sea^32^. In addition, the high impact of fishing in the area has been shown by several analyses, indicating that most of the stocks are fully exploited or overexploited^33,34^. Climate change is also strongly affecting Mediterranean marine biota and ecosystems^35,36^, mainly due to substantial temperature increases^37,38^. In fact, the Mediterranean is under a process of “meridionalization” and “tropicalization” of the northern and southern sectors, respectively, mainly due to the northward expansion of native thermophilic species and the introduction of (mainly tropical) alien species through the Suez Canal and the Strait of Gibraltar^39,40^. In addition, the Mediterranean is being altered by other anthropogenic activities such as habitat loss and degradation, pollution, and eutrophication, making the Mediterranean Sea a hotspot of global change^41,42^.

Within this context, the marine ecosystem of the Israeli Mediterranean coast, located in the eastern part of the basin, has been altered in recent decades mainly due to species invasions, unsustainable fishing activities, and increasing water temperature^29,43,44^. As a result, great changes in its biodiversity and functioning have occurred^29,30,45,46^. The importance of each stressor has rarely been investigated, and available studies suggest a general strong impact of increasing sea water temperature and more specific impacts of fishing activities and alien species^36,46^.

Recently, new fishing regulations took effect in the Israeli Mediterranean continental shelf (hereafter referred to as ICS), which includes a reduction in fishing effort for several fleets with the aim of recovering fish stocks. However, it is expected that the rate of invasion and the impact of alien species and climate change will increase in the future due to the recent enlargement of the Suez Canal and sea warming^47,48^.

In this study, we used a temporally dynamic food web model of the ICS ecosystem^49^, previously constructed and fitted to available time series of observational data from 1994 to 2010^46^, to assess potential future ecological effects of different global change scenarios. These scenarios included different fisheries management alternatives, sea warming following IPCC (Intergovernmental Panel on Climate Change) projections and projected increases in the biomass of alien species over the next 50 years (2010–2060).

## Results

### Baseline scenario

**Figure 1:**
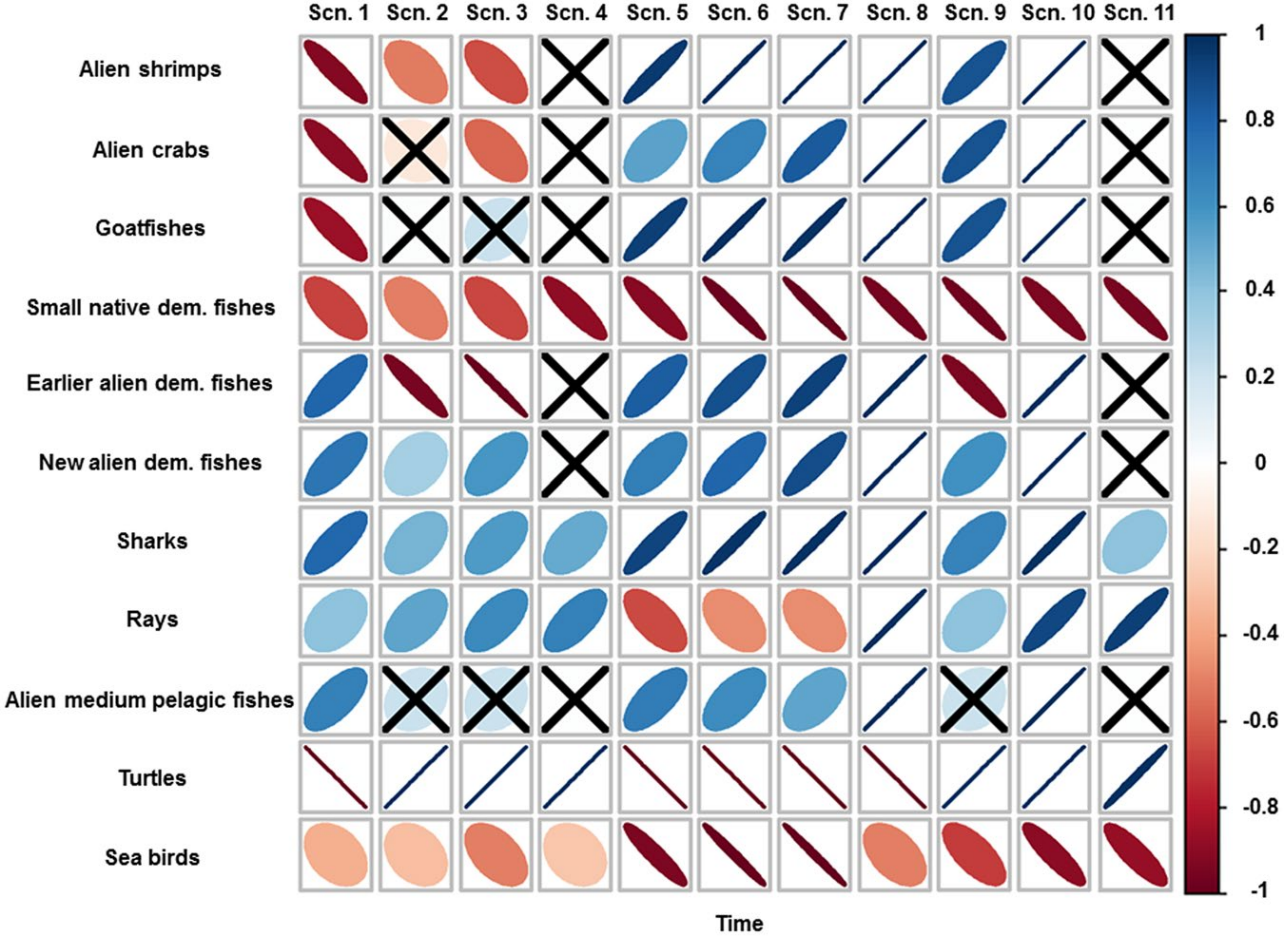
Spearman’s rank correlation between selected biomasses of functional groups and time for the ten future scenarios (Table 1). Positive correlations are in blue and negative correlations in red. Legend colour shows the correlation coefficient and its correspondent colour gradient. Colour intensity and the size of the ellipses are proportional to the correlation coefficients, with more diffused and wider ellipses representing lower correlation strengths. When the indicator is non-significant (>0.05), it is represented with an “X” symbol.

**Figure 2:**
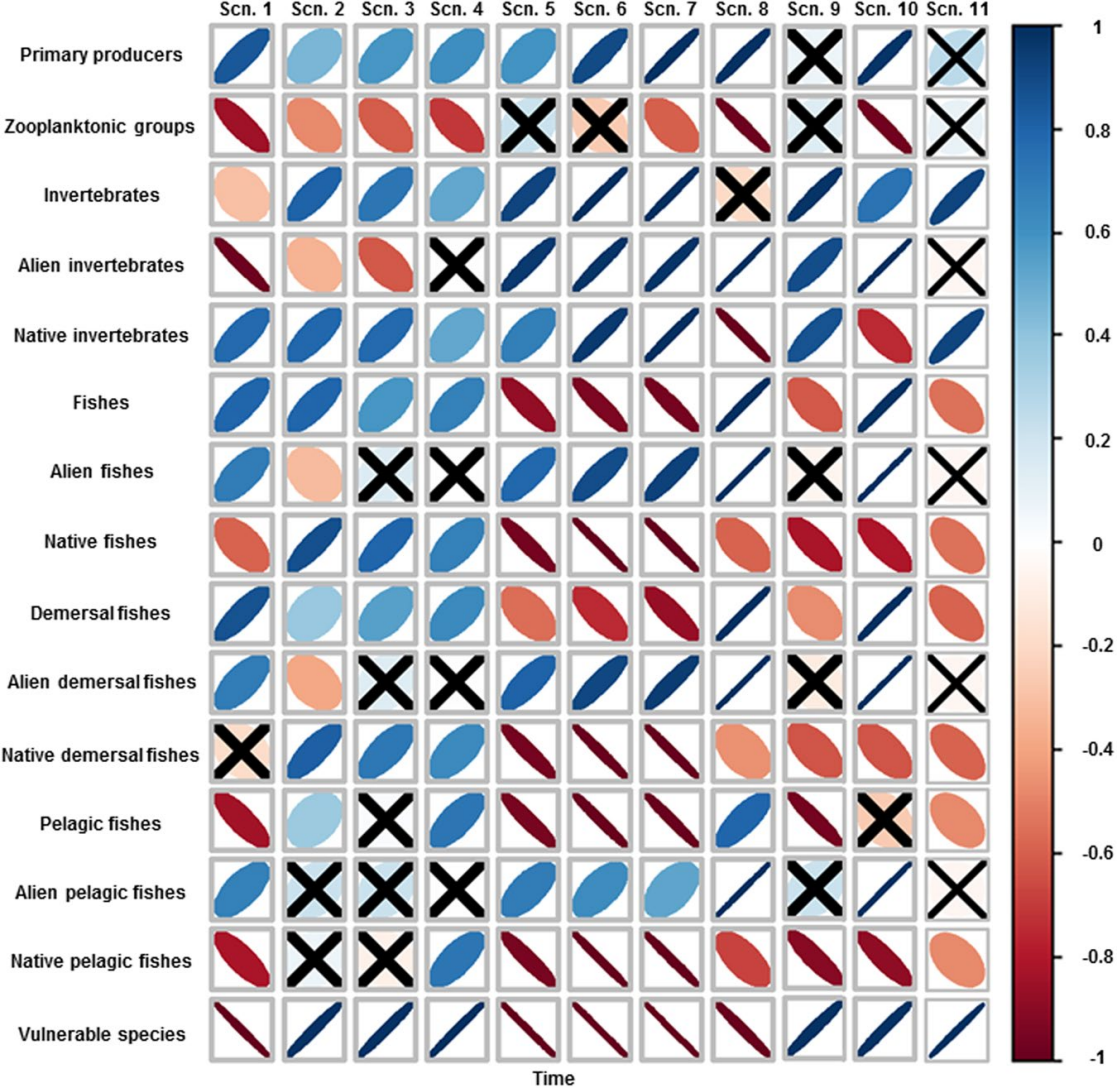
Spearman’s rank correlation between the biomass of aggregated groups and time for the ten future scenarios (Table 1). Positive correlations are in blue and negative correlations in red. Legend colour shows the correlation coefficient and its correspondent colour gradient. Colour intensity and the size of the ellipses are proportional to the correlation coefficients, with more diffused and wider ellipses representing lower correlation strengths. When the indicator is non-significant (>0.05), it is represented with an “X” symbol.

**Figure 3:**
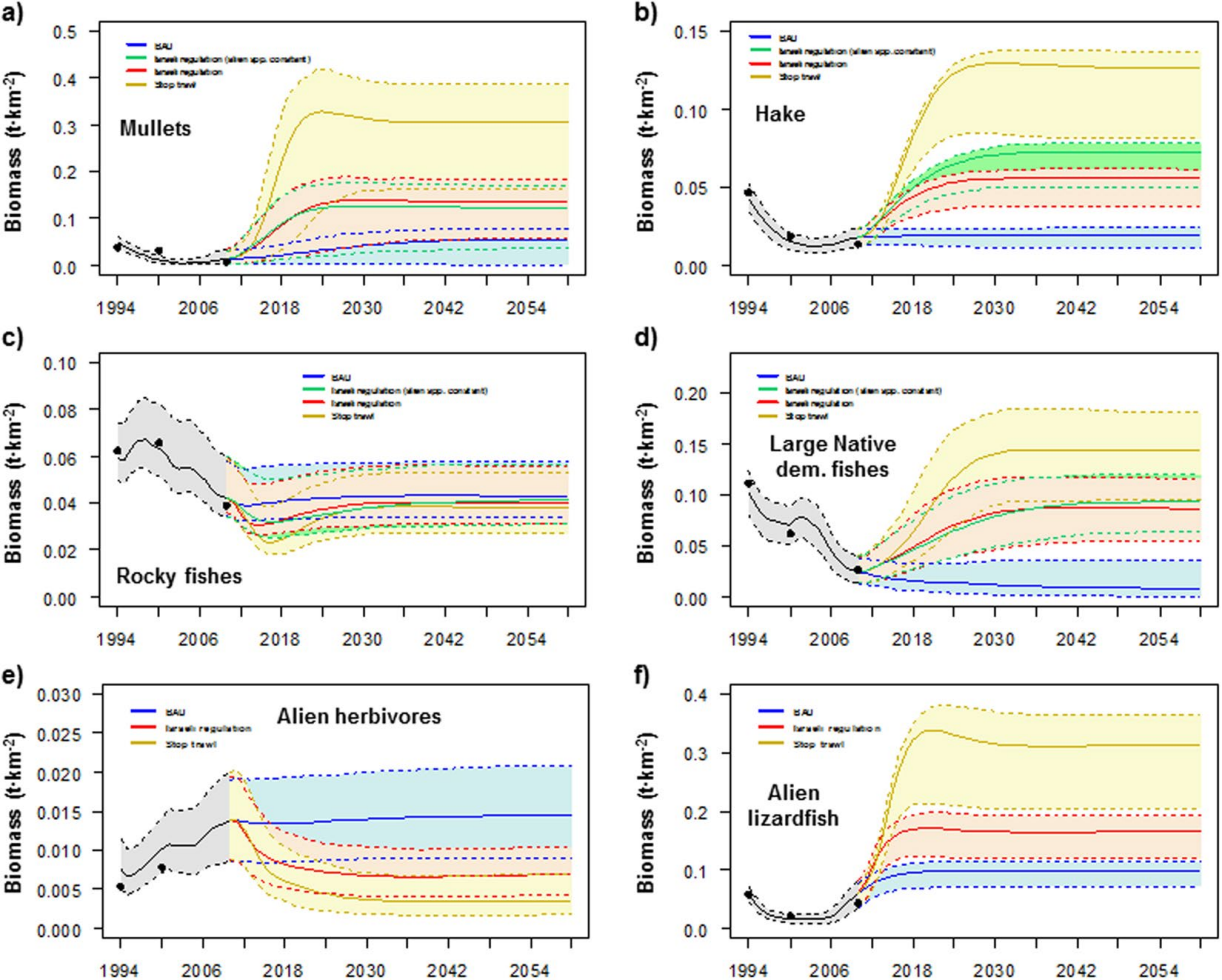
Comparison between the predicted (solid lines) and observed (dots) time series of biomass (t·km^2^), and scenarios results for (**a**) mullets, (**b**) hake, (**c**) rocky fishes, (**d**) large native demersal fishes, (**e**) alien herbivores and (**f**) alien lizardfish under different future scenarios of fishing for the Israeli Mediterranean continental shelf (ICS) ecosystem model for the period 1994–2060. Black line represents historical model predictions and coloured lines represent different scenarios. Shadows represent the 5% and 95% percentiles obtained using the Monte Carlo routine.

Under the baseline simulation (Scn1), the model predicted a decreasing biomass trend over time for the biomass of several groups (Figs 1 and 2). Alien invertebrates significantly decreased (Fig. 2), due to the depletion of alien crabs and shrimps (Fig. 1). Other medium trophic level organisms, such as goatfishes and small native demersal fishes, suffered significant large declines (Fig. 1). These decreases were due to the increase of various predators and competitors (for trophic interactions, see Figure S2b (hereafter referenced only as Figure S2b) and current negative impacts of sea warming. For example, small native demersal fishes decreased due to the increase of competitors such as earlier and new alien demersal fishes (Figs S2b and 1), the increasing predation of alien lizardfish (Fig. 3f) and the negative impact of current SST. The model also showed a significant large decline of large demersal native fishes due to their overexploitation (Fig. 3d). In addition, vulnerable species such as turtles and seabirds were projected to significantly decrease (Figs 1 and 2), due to the notable impact of fishing activities on their populations (Figure S2b).

In contrast, the model predicted significant large increases in alien fishes (both demersal and pelagic) (Fig. 2), such as earlier and new alien demersal fishes, alien lizardfish and alien medium pelagic fishes (Figs 1 and 3e). This may be due to their earlier overexploitation prior to the reduction in fishing effort between 2007 and 2010, which is mainly due to a recent decreasing activity of trawl fleet (the most important fleet in the area). This follows current biomass increases due to possible empty niches and the depletion of native competitors (Figure S2b). Mullets (Fig. 3a), sharks and rays (Fig. 1) significantly increased over time. This may be due to the decline in the fishing effort between 2007 and 2010.

**Figure 4:**
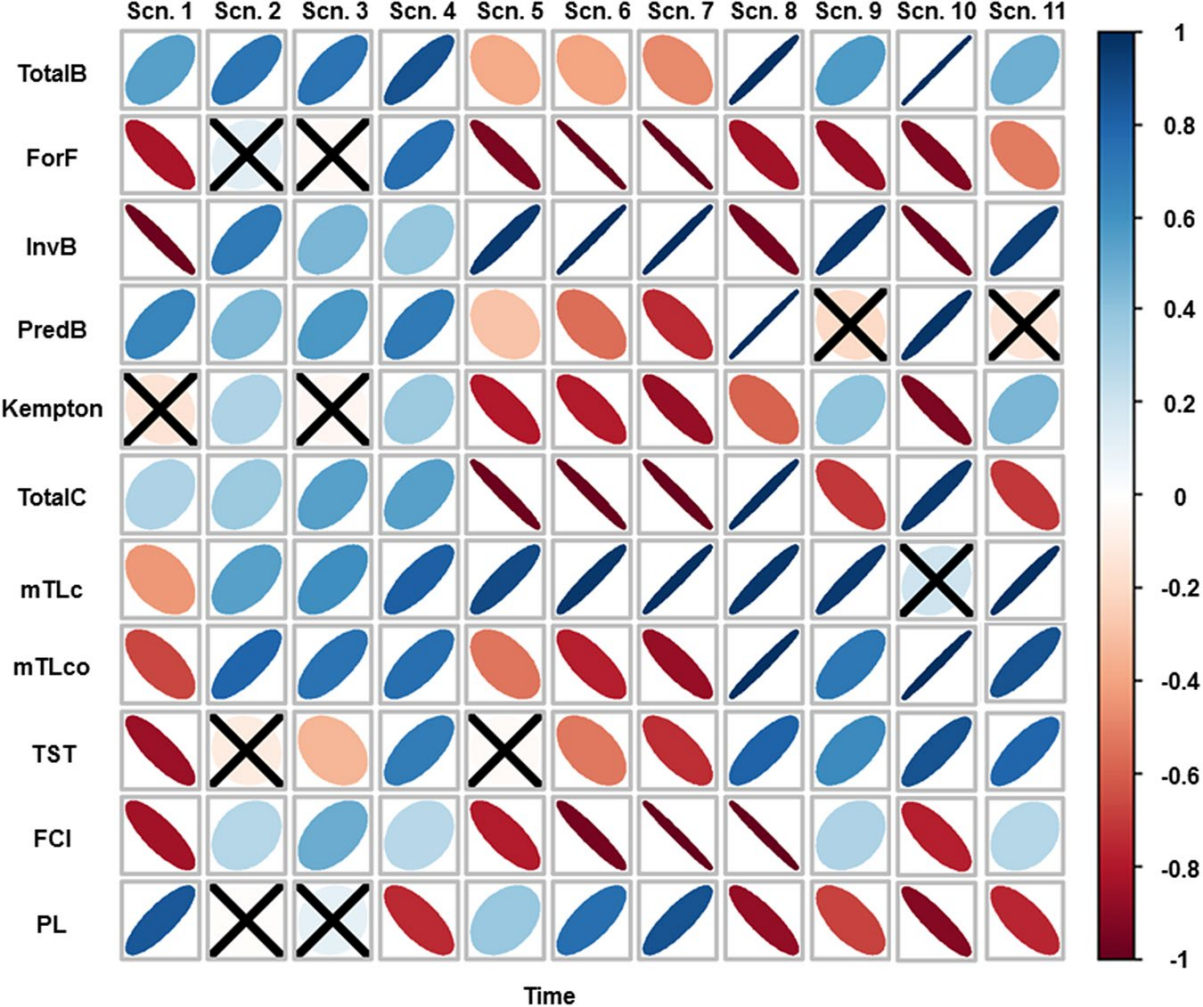
Spearman’s rank correlation between the ecological indicators analysed and time for the ten scenarios (Table 1). Positive correlations are in blue and negative correlations in red. Legend colour shows the correlation coefficient and its correspondent colour gradient. Colour intensity and the size of the ellipses are proportional to the correlation coefficients, with more diffused and wider ellipses representing lower correlation strengths. When the indicator is non-significant (>0.05), it is represented with an “X” symbol. TotalB = Total biomass (t·km^−2^); ForF = Forage fish (t·km^−2^); InvB = Invertebrate biomass (t·km^−2^); PredB = Predatory biomass (t·km^−2^); Kempton = Kempton’s index; TotalC = Total catch (t·km^−2^·year^−1^); mTLco = mean Trophic Level of the community; mTLc = mean Trophic Level of the catches; TST = Total System Throughput (t·km^−2^·year^−1^); FCI = Finn’s Cycling Index (%); PL = Path length.

Within this scenario, forage fish and invertebrate biomass decreased significantly with time while predatory biomass and total catch significantly increased over time (Fig. 4). Community indicators, such as mTLco and mTLc, and indicators related to ecosystem development theory such as TST and FCI significantly decreased with time, while PL significantly increased (Fig. 4).

### Fishing scenarios

Under scenarios that only included changes (decreases) in fishing effort (Scn2 and Scn3), the model predicted mixed trends with both significant large decreases and increases in medium trophic level groups and significant large increases in high trophic level groups (Fig. 1). For example, alien invertebrates significantly decreased while vulnerable species significantly increased (Fig. 2). Alien shrimps, small native demersal fishes, earlier alien demersal fishes and alien herbivores significantly decreased over time (Figs 1 and 3e). This is due to the increasing predation mortality as a consequence of the recovery of top predators (Figure S2b) and also, in some cases, a result of negative impacts of sea warming. In addition, the model predicted a significant decrease of sea birds due to the fewer discards caused by the reduction of the trawl fleet (Fig. 1). In contrast, the model predicted significant large increases of top predators, such as hake, large native demersal fishes, alien lizardfish, demersal sharks and rays and skates (Figs 1 and 3b,d,f). The model also showed increasing trends for mullets, new alien demersal fishes and turtles (Figs 1 and 3a), due to the reduction in fishing effort. Most of these trends were exacerbated in Scn3, with the closure of the trawl fleet. For example, the model predicted major and faster recoveries for mullets, hake, large native demersal fishes and alien lizardfish (Fig. 3a,b,d,f), while alien shrimps, small native demersal fishes, earlier alien demersal fishes and sea birds had stronger negative impacts (Fig. 1).

Under Scn4, which assessed the impacts of the new fishing regulations while keeping the biomass of alien species constant, the model showed important effects of alien species. For example, hake and large native demersal fish presented better recoveries than in Scn2 (Fig. 3b,d). For hake, this may be due to competition for resources with alien lizardfish, while for large native demersal fishes it may be due to a higher abundance of their key prey, such as rocky fishes, small native demersal fishes and earlier alien demersal fishes.

Within these three scenarios (Scn2, Scn3 and Scn4), most of the ecological indicators presented significant increasing trends (Fig. 4). For example, total biomass, invertebrate biomass, predatory biomass and total catch showed significant increasing trends (Fig. 4). In addition, mTLco and mTLc significantly increased (Fig. 4). FCI significantly increased in all scenarios while PL had non-significant trends in Scn2 and Scn3 and decreased in Scn4 (Fig. 4).

### Sea warming scenarios

Under scenarios of sea warming (Scn5, Scn6 and Scn7), the model predicted different responses of species to rising SST (Fig. 1). The model showed significant increases of alien invertebrates and alien fishes (both demersal and pelagic), while native fishes (both demersal and pelagic) and vulnerable species decreased (Fig. 2). These trends were exacerbated as temperature increased (Figs 1 and 2).

**Figure 5:**
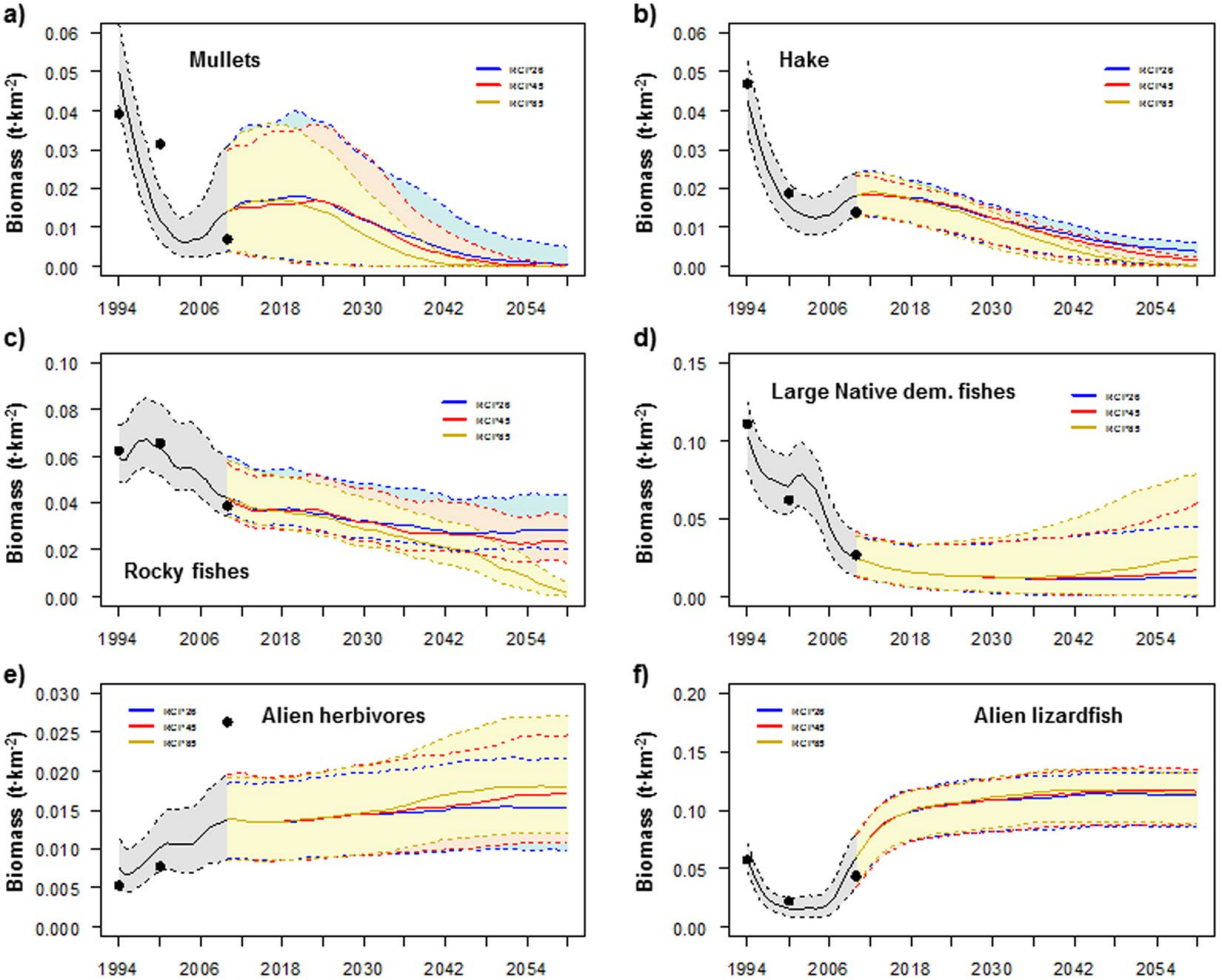
Comparison between the predicted (solid lines) and observed (dots) time series of biomass (t·km^2^), and scenarios results for (**a**) mullets, (**b**) hake, (**c**) rocky fishes, (**d**) large native demersal fishes, (**e**) alien herbivores and (**f**) alien lizardfish under different future scenarios of climate change for the Israeli Mediterranean continental shelf (ICS) ecosystem model for the period 1994–2060. Black line represents historical model predictions and coloured lines represent different scenarios. Shadows represent the 5% and 95% percentiles obtained using the Monte Carlo routine.

For specific groups, the model predicted significant increasing trends for alien shrimps, alien crabs, goatfishes, earlier and new alien demersal fishes and sharks (Fig. 1). These increases may be due to the depletion of competitors and predators (Figure S2b). In contrast, small native demersal fishes declined due to unfavourable thermal conditions, and rays and skates were projected to strongly decline (Fig. 1). A total collapse of mullets was predicted under the intermediate and worst IPCC projections (Fig. 5a), while hake and rocky fishes were predicted to be almost depleted in the worst case of sea warming (Fig. 5b,c). Large native demersal fishes were projected to be positively impacted as temperature increases (Fig. 5d), although they showed negative trends due to their overexploitation. Alien herbivores and alien lizardfish biomass significantly increased in all climate scenarios, with major increases as temperature rose except for the alien lizardfish in the worst-case scenario (Fig. 5e,f)

Within these scenarios, we observed significant decreasing trends for most of the ecological indicators, with stronger correlations as temperature increased (Fig. 4). However, invertebrate biomass, mTLc and PL showed increasing trends (Fig. 4).

### Alien species scenario

Under the scenario that assessed the impact of alien species forced to follow current biomass trends (Scn8), the model predicted strong impacts on the food web (Figs 1 and 2). Within this scenario, native invertebrates, native fishes (both demersal and pelagic) and vulnerable species declined significantly (Fig. 2).

**Figure 6:**
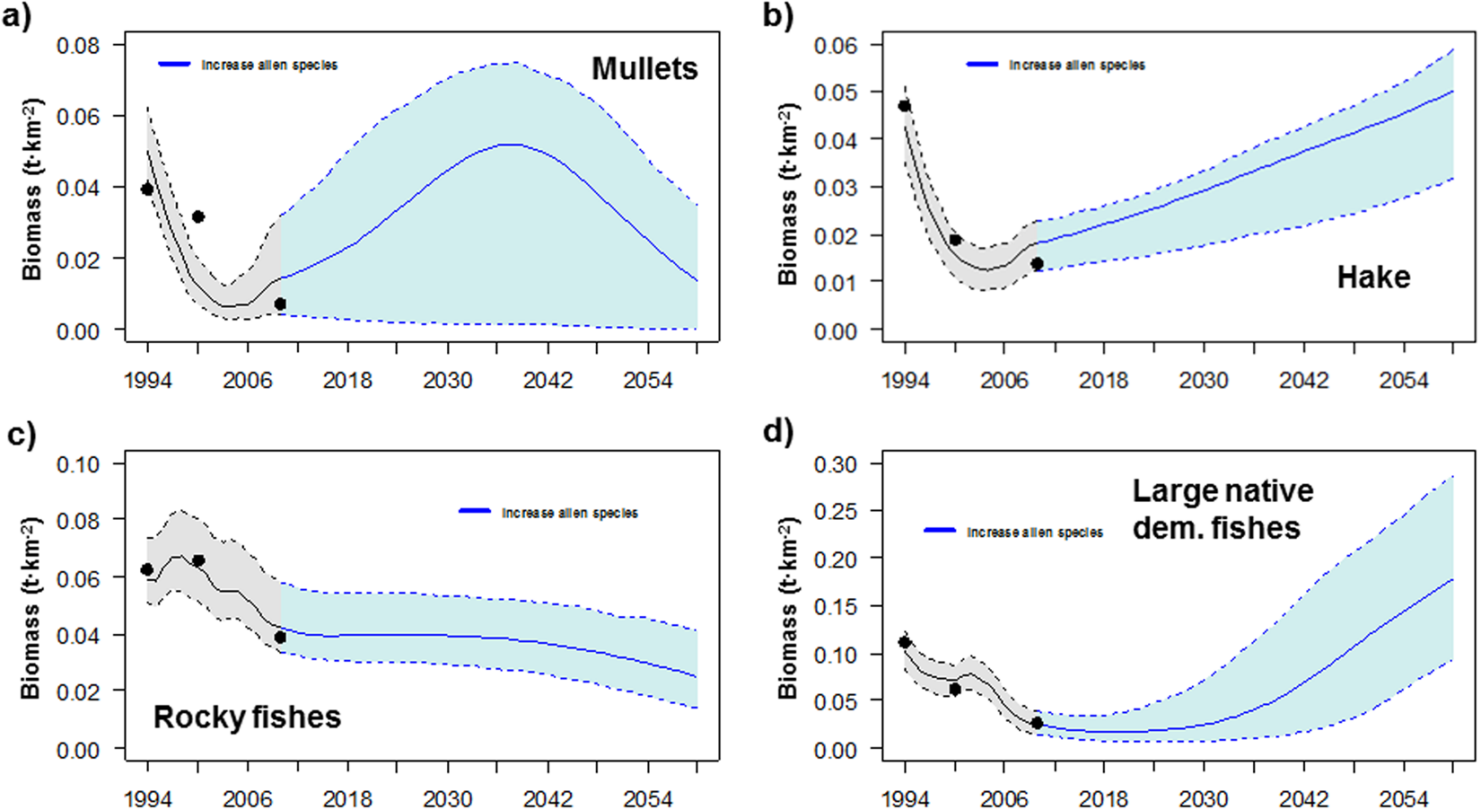
Comparison between the predicted (solid lines) and observed (dots) time series of biomass (t·km^2^), and scenarios results for (**a**) mullets, (**b**) hake, (**c**) rocky fishes (or small native demersal fishes) and (**d**) large native demersal fishes under the future scenario of increasing the biomass of alien species for the Israeli Mediterranean continental shelf (ICS) ecosystem model for the period 1994–2060. Black line represents historical model predictions and coloured lines represent different scenarios. Shadows represent the 5% and 95% percentiles obtained using the Monte Carlo routine.

For specific groups, the model predicted significant decreases of small native demersal fishes due to current thermal conditions and increasing predation mortality and competition (Figure S2b). Similarly, turtles and sea birds declined due to a decline of their main prey (Figs S2b and 4). Mullets were predicted to be slightly negatively impacted, due to their initial recovery as a result of the decreasing fishing effort in 2007–2010 and the negative impacts of alien species (Figs S2b and 6a). Rocky fishes declined significantly, due to a higher abundance of competitors and predators (Figs S2b and 6c). In contrast, hake and large native demersal fishes (Fig. 6b,d) as well as demersal sharks and rays and skates (Fig. 1) significantly increased. This may be due to reduced fishing activities and a higher abundance of alien prey (Figure S2b), although native prey exhibited opposite trends (Fig. 2).

Under this scenario, total biomass, predatory biomass and total catch significantly increased (Fig. 4). In contrast, forage fish, invertebrate biomass and Kempton’s index significantly decreased (Fig. 4). FCI and PL were projected to decline significantly, while TST increased (Fig. 4).

### Cumulative scenarios

**Figure 7:**
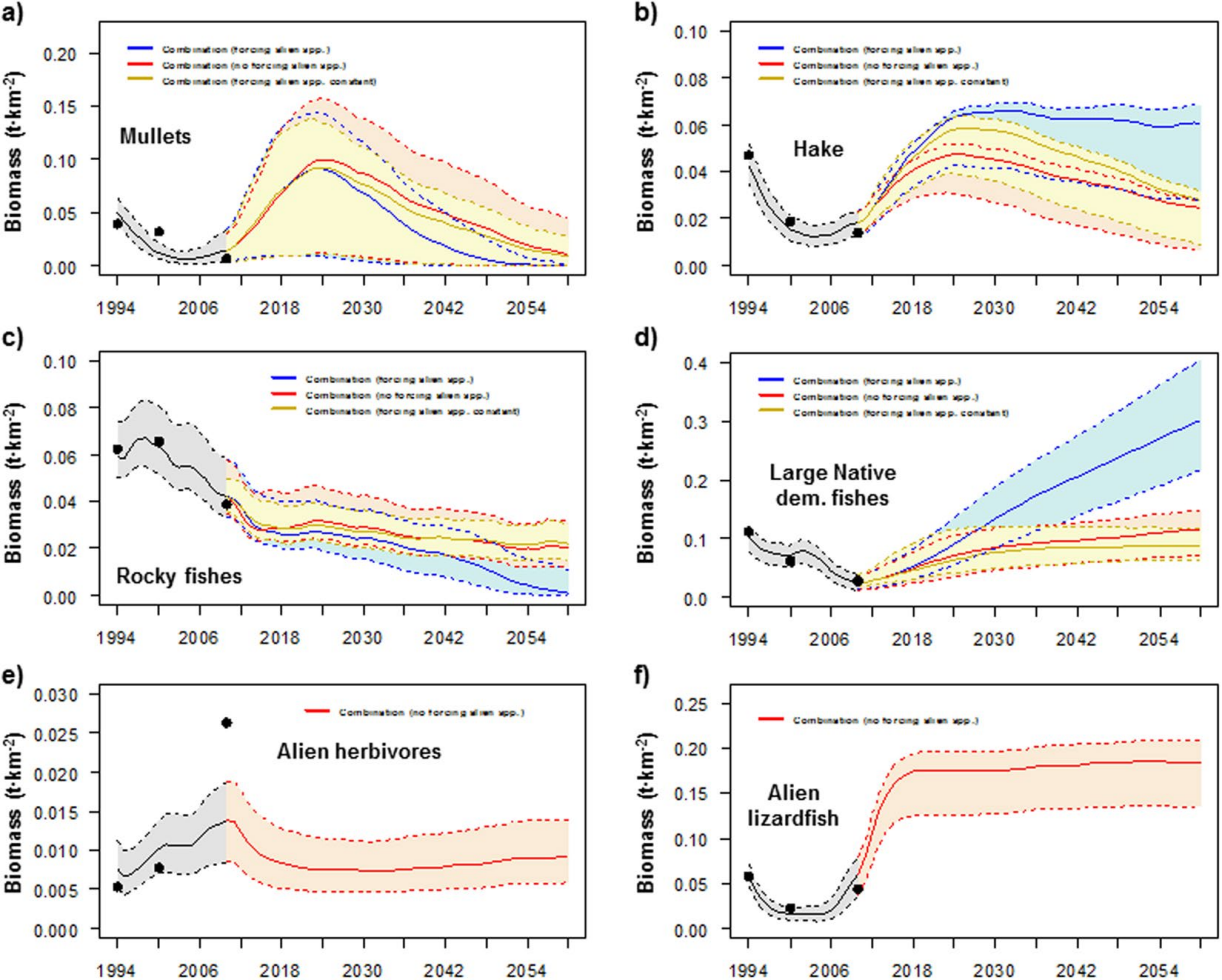
Comparison between the predicted (solid lines) and observed (dots) time series of biomass (t·km^2^), and scenarios results for (**a**) mullets, (**b**) hake, (**c**) rocky fishes, (**d**) large native demersal fishes, (**e**) alien herbivores and (**f**) alien lizardfish under different future scenarios of a combination of stressors for the Israeli Mediterranean continental shelf (ICS) ecosystem model for the period 1994–2060. Black line represents historical model predictions and coloured lines represent different scenarios. Shadows represent the 5% and 95% percentiles obtained using the Monte Carlo routine. Scenarios that include forcing of the biomass are not shown.

When assessing the cumulative effects of new Israeli fishing regulations and an intermediate scenario of an increase in SST, while alien species biomass was not forced (Scn9), the model projected biomass increases for native invertebrates, alien groups (both invertebrates and fishes) and vulnerable species, while the biomass of native fishes (both demersal and pelagic) significantly decreased (Fig. 2). For specific groups, the biomass of some significantly increased such as alien shrimps and crabs, goatfishes, new alien demersal fishes, demersal sharks, rays and skates, and turtles (Fig. 1). In addition, significant increases were observed for hake, large demersal fishes and alien lizardfish, but their recoveries were of a lower magnitude than Scn10 due to the limitation of alien prey (Fig. 7b,d,f). In fact, hake declined at the end of the simulation due to sea warming (Fig. 7b). In contrast, the biomass of small native demersal fishes, earlier alien demersal fishes and sea birds significantly decreased (Fig. 1). In addition, the model predicted significant declines in mullets and rocky fishes (Fig. 7a,c), although they showed better trajectories than Scn10, due to lower impacts of alien species. Alien herbivores also declined (Fig. 7e), due to recoveries of predators (both native and alien) (Figure S2b).

Under this scenario, total biomass, invertebrate biomass, mTLc and mTLco significantly increased, while forage fish and total catch significantly declined (Fig. 4). TST and FCI were projected to increase, while PL declined (Fig. 4).

When assessing the cumulative effects of the new Israeli fishing regulations, the intermediate scenario of sea warming and an increase in alien biomass following current trends (Scn10), the model predicted a significant decreasing pattern of native invertebrates and native fishes (both demersal and pelagic), while vulnerable species significantly increased (Fig. 2). Several groups that were negatively affected included small native demersal fishes and sea birds (Fig. 1). In addition, the model predicted a near collapse of mullets (Fig. 7a), despite the reduction of fishing effort, and a significant decline of rocky fishes (Fig. 7c). In contrast, demersal sharks, rays and skates and turtles significantly increased (Fig. 1). In addition, hake and large native demersal fishes were predicted to increase (Fig. 7b,d), mainly due to reduced fishing effort and a higher abundance of alien prey (Figure S2b), although native prey significantly declined and there was negative impact of SST on hake.

Within this scenario, forage fish, invertebrate biomass and Kempton’s index significantly decreased, while predatory biomass, total catch and mTLco significantly increased (Fig. 4). FCI and PL were projected to decline significantly, while TST increased (Fig. 4).

Finally, under the assessment of the cumulative impact of the new Israeli fishing regulations and the intermediate scenario of sea warming, while keeping the biomass of alien species constant (Scn11), the model highlighted the important effects of alien species. For example, native invertebrates increased more than in Scn9 and native fishes decreased less than Scn9 and Scn10 (Fig. 2). For specific groups, small native demersal fishes decreased less than Scn9 and Scn10 (Fig. 1). Hake presented a better trajectory than Scn9 (Fig. 7b). This could be due to a less competition for resources with alien lizardfish, which biomass was kept at constant population levels. However, it presented a worst trajectory than Scn10 (Fig. 7), which could be due less prey availability. On the other hand, large native demersal fishes presented a worse trajectory than Scn9 and Scn10 (Fig. 7d), which could be also due to less prey availability. Mullets and rocky fishes presented similar trajectories than Scn9 (Fig. 7a,c), which may be related to similar predation rates and competition for resources in both scenarios.

Within this scenario, ecological indicators presented similar trends to Scn9 (Fig. 4). In several cases slightly better trends than Scn9 were observed, such as in forage fish, Kempton’s index, mTLc and mTLco, while total catch and PL presented slightly worse trends than Scn9 (Fig. 4).

### Common patterns of future scenarios

In general, primary producers increased in most of the scenarios (Fig. 2). This can be attributed to the decrease of zooplanktonic groups in most of the scenarios (Fig. 2), which is due to increased predation on these groups. Alien invertebrates decreased in scenarios that only fishing reductions were applied while increasing in the other scenarios (Fig. 2). This can be attributed to increasing predation and decreasing predation and competition, respectively. Alien fishes increased in all scenarios due to reductions of competition and predators except in Scn2 (Fig. 2), where there is a large decrease in earlier alien demersal fishes attributed to higher predation rates (Fig. 1). New alien demersal fishes increased in all the scenarios, which may due to fishing reductions and/or the decrease of competition (both native and alien groups). Native fishes decreased in all scenarios except those scenarios where only fishing reductions were applied (Fig. 1). Specifically, small native demersal fishes decreased in all scenarios. This general pattern is due to unfavorable thermal conditions, while for specific scenarios we can add increasing predation (fishing reductions) and competition for resources (alien species scenarios) or both (cumulative scenarios) as the main drivers of the ecological patterns. Vulnerable species increased in all scenarios that implied reductions in fishing activities (Fig. 2), although sea birds decreased in all scenarios (Fig. 1).

## Discussion

In the current context of global change and ecological crisis, there is an increasing demand for approaches that can forecast potential impacts of human stressors, in addition to environmental pressures^16^. In this study, we used a temporal dynamic food web model for the Israeli Mediterranean continental shelf that accounted for different environmental and human impacts, such as sea warming, fisheries and alien species, to assess potential futures of marine resources and ecosystem conditions of the Eastern Mediterranean Sea. Despite several limitations, this study represents to our knowledge the first attempt to evaluate potential impacts of future conditions in the Eastern Mediterranean Sea in an ecosystem context combining different global change stressors.

Our results highlight that under current conditions (the baseline scenario) several species will remain depleted or even greatly decline, due to unfavourable thermal conditions, increasing impacts of alien species, and unsustainable fishing activities. Meanwhile, alien groups will continue to increase in abundance, as many of these species have higher thermal tolerances. This general degradation of the system is also captured by the decline of ecological indicators linked with ecosystem condition, such as mTLc, mTLco and FCI. These results are in line with Corrales, *et al*.^46^, where results indicated a historical degradation pattern of the food web over the last two decades (1990–2010) due to the impacts of alien species, climate change and fishing. However, other ecological indicators increased, such as total biomass, predatory biomass and PL. This could be due to the fact that reductions of native species in terms of biomass and path lengths are compensated by the increase of alien species.

In contrast, when fishing effort for several fleets was reduced, our results highlighted a potential restoration of several exploited groups including commercially important species such as hake, mullets and large native demersal fishes, and some vulnerable species such as sharks and rays and skates. Alien groups (fish and crustaceans) were negatively impacted, mainly due to the recovery of predators, while native groups were positively affected. This overall improvement of some marine resources was captured by several ecological indicators that showed a trend of increasing values, such as the predatory biomass, Kempton’s Index, mTLc, mTLco and FCI.

Fishing has been identified as one of the main stressors on marine ecosystems^50,51^, and studies have shown the potential benefits of fishing reduction^52,53^. Our results highlighted the benefits of reducing fishing activities on the exploited marine organisms and ecosystem in the Eastern Mediterranean Sea, and support the call for a reduction in fishing capacity and exploitation levels worldwide if marine resources are to recover^53,54^.

The scenarios of sea warming showed potential detrimental impacts on the food web, with the impacts becoming greater as temperature increased. Within these scenarios, native species were negatively impacted, and we observed some collapses, while alien species were favoured. In line with this, several ecological indicators, including Kempton’s Index, mTLco and FCI suggested a potential degradation of the ecosystem. Predicted collapses of some native species in this study may not indicate a total collapse of the species in the Eastern Mediterranean Sea, but may indicate that if these species are to persist in the ecosystem, they may have to migrate to northern areas or to deeper and cooler waters outside of the modelled area, or they will have to adapt. Shifts in species distributions (latitudinal and bathymetric) in relation to climate change have been observed and predicted in many areas of the world^55–57^. Bathymetric shifts and species collapses have been observed recently in the study area associated with sea warming and the proliferation of alien species^29,58^. In addition, several studies have predicted important changes in species distributions due to sea warming in the Mediterranean Sea^48,59^. In fact, the increasing importance of alien species (thermophilic biota) concurrent with sea warming has led to the tropicalization of the Mediterranean biota^40^.

Our projections of the impact of sea warming present some limitations. For example, the temperature response/preferences used in our study are subject to uncertainty, as they came from a global database (AquaMaps)^60^, although we did incorporate local knowledge to adapt the global responses to local conditions (see Corrales, *et al*.^46^ for more details). In addition, due to the lack of information on the responses to the explanatory variable change, our model did not incorporate salinity, which has been suggested as an important environmental factor in the study area^61^. Also, other impacts of climate change were not considered. For example, ocean acidification, which mainly acts on invertebrates and basal species, can have strong impacts on the food web^62,63^. Furthermore, our model does not account for the possible acclimatization, selection, and adaptation of species to climate change. Correctly predicting the impacts of climate change on marine organisms and ecosystems remains challenging due to a general lack of knowledge about the capacity of organisms to adapt to rapid climate change^64^. In addition, our model is a temporal-dynamic representation of the ecosystem and does not explicitly incorporate spatial dynamics (such as movement of species) and therefore the potential movement of species to deeper waters or latitudinal (northward) shifts are not captured. Within this context, the new habitat foraging capacity model of the spatial-temporal module of EwE, Ecospace^65,66^, has provided a step forward for temporal-spatial modelling by combining species distribution and food web models. As new information becomes available, our modelling exercise should be updated and improved, so model predictions would become increasingly valuable for understanding cumulative impacts within a spatial-temporal dynamic framework.

Our results highlighted the potential negative impacts of alien species on marine species and food webs, either when extrapolating current trends to the future, or when allowing EwE to predict their future abundance. Alien species proliferation causes the collapse of small native demersal fishes and a degradation pattern in the food web, as shown by different ecological indicators (i.e., predatory biomass, Kempton’s index, mTLco, FCI and PL). Biological invasions are considered a major threat to local biodiversity^28,67^. Although no complete extinctions have yet been reported in the Mediterranean Sea as a direct result of alien species, there are many examples of sudden declines and local extirpations of native species concurrent with the proliferation of alien species^29,68^.

It is important to note that our model has a limited capacity to assess the impacts of alien species. Our study only considers alien fish and crustacean (shrimps and crabs) species, since for other groups no information was available to be considered within our temporal modelling approach^69^. However, the invasion of other organisms seems to be of the same magnitude or even greater^69,70^. In addition, the information about pelagic fishes were limited and the definition of small and medium pelagic fishes groups within the model includes both native and alien species^46^. Finally, several new alien species have invaded the Eastern Mediterranean Sea in recent years and were not included in the model^31,32^. One of these species, the lionfish (*Pterois miles*), has alarmed the scientific community, arriving in the Mediterranean Sea in 1991^71^ but not recorded again until 2012^72^. This species has had detrimental effects on invaded ecosystems, such as the Caribbean Sea^73^. It is expected that the current and future enlargement of the Suez Canal and future sea warming will allow the invasion of more species^74^, and that the Eastern Mediterranean Sea can become an extension of the Red Sea in terms of species composition, even including reef building corals^75,76^.

Under cumulative stressor scenarios, our study showed that the beneficial effects of fisheries reduction could be dampened by the combined impacts of sea warming and alien species. For example, mullets, hake and predators in general may not recover if sea warming and alien species impacts are also at play. These results highlight the need to include stressors other than fisheries, such as climate change and biological invasions, in the assessment of risk and the implementation of an ecosystem-based management approach to correctly assess the future of marine ecosystems. Serpetti, *et al*.^26^, using an EwE model on the west coast of Scotland, highlighted that ocean warming could jeopardize sustainable fisheries practices in the future. Our results are complementary to this study and suggest that regional and global scale impacts such as biological invasions and sea warming can impair, or at least limit, the outputs of local fisheries management measures.

There is an increasing need to identify and quantify the biophysical thresholds that must not be exceeded, so as to prevent catastrophic shifts in ecosystems. Catastrophic shifts can be defined as persistent and substantial reorganizations of the structure and functioning of ecosystems and from which their recovery is difficult or impossible^77,78^. The boundaries of several processes (e.g., climate change and biodiversity loss) define the “safe operating space” for humanity^78^. However, crossing certain boundaries may take the ecosystem beyond its “safe operating space”, where the risk of unpredictable and damaging change is very high. Our results highlighted the fact that a reduction in fishing activities promotes the resilience of some species to climate change and the impacts of alien species in the Eastern Mediterranean Sea, with resilience defined as the capacity of species and ecosystems to resist and absorb disturbance and their ability to recover^79,80^. In addition, some native species reacted better to reduced fishing activities when alien species were maintained at constant levels in the absence and presence of sea warming. However, once a boundary is crossed, a species can collapse. In our study, this is the case for mullets and hake. These species have been severely impacted in recent decades by fishing activities, alien species (goatfishes and alien lizardfish, respectively), and sea warming^46,68,81–83^. In the cumulative impact scenarios, these functional groups initially benefited from reduced fishing effort. However, once the boundary of thermal tolerance was crossed, mullets and hake decreased notably. When we forced an increase in alien species biomass, in addition to sea warming, mullets collapsed due to the additional effects of predation and competition, while hake biomass remained almost constant due to the higher abundance of prey. Our study illustrates that complex dynamics between environmental and ecological processes may interact in the future and it is essential to take them into account.

In recent decades, human activities have exponentially increased^1^. These include local stressors such as overfishing, habitat destruction and pollution, and regional and global stressors, such as biological invasions and climate change. Such anthropogenic effects impose large impacts on marine organisms and ecosystems, affecting ecosystem structure and services^4,67,84^. Organisms and ecosystems already stressed by fishing are more vulnerable to further impacts such as climate change and biological invasions^55,85^. As temperature will increase in the future and options for the management of ocean warming are limited at the local and regional scale, reducing local and regional threats such as overexploitation and biological invasions, may be one of the solutions to promoting resilience to climate change, ensuring the capacity to exploit marine resources safely and preserving ecosystem functions and services^57,86^.

Different management actions have been used for reducing the impacts of fisheries, including, among others, the establishment of catch limits, fishing effort reductions, increasing gear selectivity and the implementation of Marine Protected Areas (MPAs)^87^. MPAs have been suggested as an effective tool to mitigate impacts of climate change and alien species^88,89^, although biological invasions have been largely disregarded in marine conservation plans^90^ and the effectiveness of MPAs in preventing invasions has been questioned^91^. The prevention of new introductions should be a priority in the development of effective policies, followed by early detection, rapid response and possible eradication of alien species^92^. In the context of our study area, some authors have suggested installing an environmental barrier in the Suez Canal, such as an hypersaline lock, since it may “reduce the likelihood of species migration through canals”^93^. In fact, “the Suez Canal had, for nearly a century, a natural salinity barrier in the form of the high salinity Bitter Lakes”^93^. In addition, although eradication is challenging, some countries have initiated eradication programs to minimize the impacts of alien species in the marine environment. For example, in Cyprus, governmental authorities encouraged fishermen to catch alien poisonous pufferfish (*Lagocephalus sceleratus*)^94^, which have detrimental effects on native biota and fisheries^95^.

Ecological indicators are quantitative measurements that provide information about key ecosystem characteristics. They are increasingly used to document ecosystem status and to track the effects of anthropogenic and environmental stressors on marine ecosystems, as well as the effectiveness of management measures; making them a valuable tool within the EBM framework^96–98^. We showed that trophic level-based indicators (mTLc and mTLco) were informative about the effects of fishing pressure, as they decreased in the baseline scenario (high fishing pressure) while increasing in all scenarios where fishing reductions were implemented. However, they exhibited opposite trends in sea warming scenarios. The predatory biomass indicator also indicated potential benefits of fishing restrictions, as well as detrimental impacts of sea warming. In addition, Kempton’s index successfully tracked fishing pressure, sea warming and impacts of alien species. Therefore, our study illustrates how several ecological indicators obtained from EwE models can be useful to assess ecosystem status^99,100^, but they may show complex trends to interpret as additional pressures to marine ecosystems are investigated.

## Material and Methods

### Study area

The Israeli Mediterranean continental shelf (ICS) ecosystem (Figure S1) is located in the Eastern Mediterranean Sea, also known as the Levantine Sea. The Levantine Sea has the hottest, most saline and most oligotrophic waters in the Mediterranean Sea^101,102^ as a result of high evaporation rates, very low riverine inputs and limited vertical mixing.

Currently, the Levantine Sea is the world’s most invaded marine ecoregion, with important effects on the food web^29,103^. In addition, it has been suggested that intense fishing pressure has jeopardized the sustainability of fishing activities^104^. Finally, the waters of the Levantine Sea are warming at higher rates than the global average^37,105^, with important effects on marine biota^36,58^.

### Overview of the modelling approach

The ecological modelling approach Ecopath with Ecosim (EwE)^106^ was used to model the study area. The EwE approach consists of three main modules: the mass-balance routine Ecopath, the time dynamic routine Ecosim and the spatial-temporal dynamic module Ecospace. For an extensive review of EwE principles, basic concepts, capabilities and limitations, see Christensen and Walters^106^ and Heymans, *et al*.^107^.

The Ecopath mass-balance model was developed using EwE version 6.5 (www.ecopath.org) to characterise the structure and functioning of the ICS and to assess the past and current impact of alien species and fishing^49^. The model covered an area of 3,725 km^2^, with coastal waters up to 200 m in depth. It represented two time periods (1990–1994 and 2008–2010), including 39 and 41 functional groups, respectively, from primary producers to top predators and considers specific groups for alien species (Figure S2a; Table S1)^49^. This model took into account the main fleets operating in the area, including bottom trawl, purse seine and artisanal fisheries, and recreational fishers. Direct and indirect trophic impacts between functional groups and fleets are shown in Figure S2b.

Based on the Ecopath model, the time dynamic module Ecosim^108^ was constructed and fitted to time series of data from 1994 to 2010. The model was used to consider the combined effect of alien species, fishing activities and changes in sea surface temperature and primary productivity^46^. Ecosim uses a set of differential equations to describe biomass dynamics, expressed as:

1 $$\frac{d{B}_{i}}{dt}={(\frac{P}{Q})}_{i}\cdot {\sum }^{}{Q}_{ji}-{\sum }^{}{Q}_{ij}+{I}_{i}-({M}_{i}+{F}_{i}+{e}_{i})\cdot {B}_{i}$$

where *dB*_*i*_*/dt* is the growth rate of group (i) during time t in terms of its biomass *B*_*i*_; *(P/Q)*_*i*_ is the net growth efficiency of group (i); *M*_*i*_ is the non-predation mortality rate; *F*_*i*_ is the fishing mortality rate; *e*_*i*_ is the emigration; and *I*_*i*_ is the immigration rate^106^. Consumption rates (*Q*_*ij*_) are calculated based on the “foraging arena” theory^109^, which divides the biomass of a prey into a vulnerable and a non-vulnerable fraction and the transfer rate or vulnerability between the two fractions determines the trophic flow between the predator and the prey. The vulnerability concept incorporates density-dependency and expresses how far a group is from its carrying capacity^106,110^. For each predator-prey interaction, consumption rates are calculated as:

2 $$Qij=\frac{{a}_{ij}\ast {v}_{ij}\ast {B}_{i}\ast {P}_{j}\ast {T}_{i}\ast {T}_{j}\ast {M}_{ij}/{D}_{j}}{{v}_{ij}+{v}_{ij}\ast {T}_{i}\ast {M}_{ij}+{a}_{ij}\ast {M}_{ij}\ast {P}_{i}\ast {T}_{j}/{D}_{j}}\ast f(En{v}_{function},t)$$

where a_ij_ is the rate of effective search for prey (i) by predator (j), v_ij_ is the vulnerability parameter, Ti represents prey relative feeding time, T_j_ is the predator relative feeding time, B_i_ is prey biomass, P_j_ is predator abundance, M_ij_ is the mediation forcing effects, and D_j_ represents effects of handing time as a limit to consumption rate^109,110^. Environmental response functions (Env_function_, t), which represents the tolerance relationship of a species to an environmental parameter (here defined with a minimum and maximum levels and the 10th and 90th preferable quantiles), can be used to account for environmental drivers that change overtime, such as temperature. The intercept between the environmental response function and the environmental driver is used to calculate a multiplier factor (f) (eq. 2), which then modifies the consumption rates of a species, or functional group, with a maximum value of 1 and declining values (and thus limiting the foraging capacity of a group) when the environmental driver deviates from the optimum values^26,66^.

A time series of nominal fishing effort from the Fisheries Department of the Ministry of Agriculture and Rural Development of Israel was used to drive the model by modifying fishing mortality on targeted groups. A time series of annual sea surface temperature (SST, upper 30 meters) from 1994 to 2010 and temperature response functions were used to drive the temporal dynamics of sensitive functional groups with available information (mostly crustaceans and fish groups)^46^. Time series of SST were obtained from the Mediterranean Forecasting System Copernicus (http://marine.copernicus.eu/). Environmental response functions, which here determine optimum temperatures and thermal tolerance, were obtained initially from AquaMaps^60^ and were modified incorporating expert local knowledge (see Corrales, *et al*.^46^ and Table S2 for further details).

### Simulation of future scenarios

We used the temporal dynamic module Ecosim to evaluate the effect of plausible future scenarios for major stressors in the area (Table 1). With the exception of the two new alien groups (new alien demersal fishes and alien medium pelagic fishes), we used the original Ecosim configuration that was fitted to the time series of data^46^. For these two new alien groups, low vulnerability values had been estimated by the model in the fitting procedure, impeding a further increase in biomass of these groups in the future. As a continuous increase in biomass of these groups is expected, we applied a high vulnerability value (*v* = 10) to them to allow a larger change in the baseline predation mortality. All future scenarios were run for 50 years, from 2010 to 2060, and included variations of different stressors (Table 1). Primary production, in the absence of information about projected potential changes, was kept constant in all the scenarios from 2010 to 2060.

**Table 1:** List of scenarios and stressor conditions.

Scenario	Name	Fishing	Temperature	Alien species
1	BAU (business as usual)	Kept at 2010 levels	Kept at 2010 level	Model predicts
2	Israeli regulation	New Israeli regulations	Kept at 2010 level	Model predicts
3	Stop trawl	New Israeli regulations + stop trawl in 3 years	Kept at 2010 level	Model predicts
4	Israeli regulation (alien spp. constant)	New Israeli regulations	Kept at 2010 level	Force (kept at 2010 levels)
5	RCP2.6	Kept at 2010 levels	Best-case	Model predicts
6	RCP4.5	Kept at 2010 levels	Intermediate	Model predicts
7	RCP8.5	Kept at 2010 levels	Worst-case	Model predicts
8	Increase alien species	Kept at 2010 levels	Kept at 2010 level	Force (increase)
9	Combination (no forcing of alien spp.)	New Israeli regulations	Intermediate	Model predicts
10	Combination (forcing of alien spp.)	New Israeli regulations	Intermediate	Force (increase)
11	Combination (forcing alien spp. constant)	New Israeli regulations	Intermediate	Force (kept at 2010 levels)

**Figure 8:**
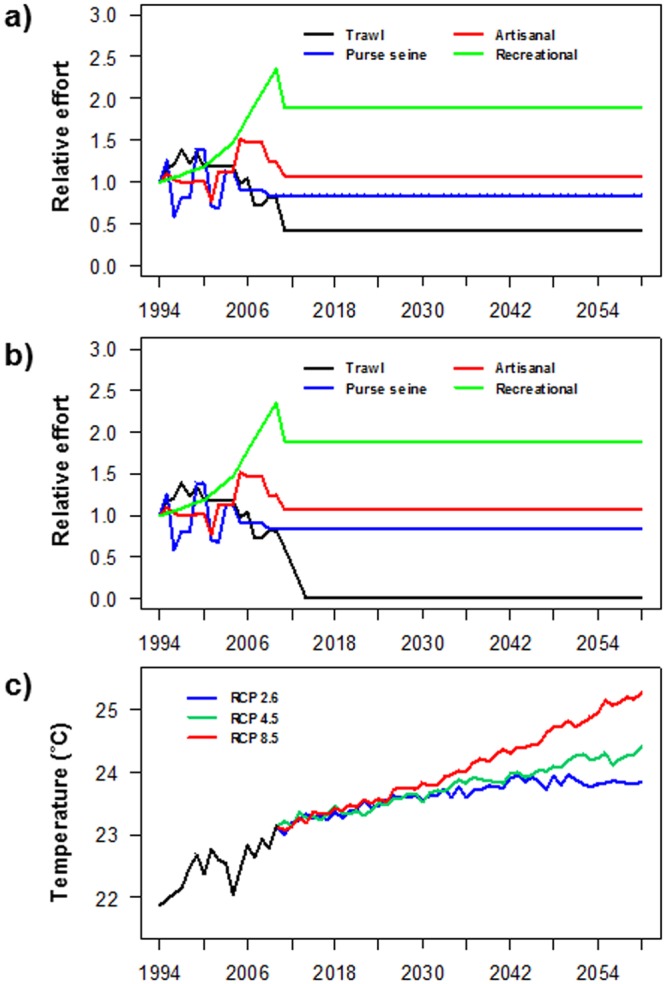
Stressors in the Israeli Mediterranean continental shelf (ICS) ecosystem for the period 1994–2060 considered in this study: (**a**) relative fishing effort by fleet as a result of the application of the new Israeli law starting in 2010 in the simulations; (**b**) relative fishing effort by fleet as a result of the application of the new Israeli law with the closure of the trawl fleet after three years of reduction from 2010; and (**c**) historical annual sea surface temperature (black line) and its projection under the three scenarios of IPCC projections.

The original configuration of the dynamic model was used as a baseline simulation (Business as usual (BAU)) (Scn1). We then assessed the impact of various fisheries management strategies while keeping constant temperature levels from 2010 to 2060. Scn2 included the new fishing regulations approved by the Fisheries Department of the Ministry of Agriculture and Rural Development of Israel in 2016. These regulations, among other components, consist of a reduction in fishing efforts for the trawling and artisanal sectors and impose restrictions on the recreational fishers. For the trawl fleet, a complete cessation of its activity between April and June was implemented. In addition, the trawl fleet in the northern part of the country is to be mostly eliminated. These two regulations were implemented in our scenario and represented a reduction in trawl effort of nearly 50% (Fig. 8a). For the artisanal fleet, a ban between April and May was implemented and implied a reduction in fishing effort of nearly 15% (Fig. 8a). For recreational fishers, the new regulation restricted their capacity to a maximum catch of 5 kg per day. In the absence of detailed data about recreational effort and being conservative, a reduction of 20% of the effort was applied (Fig. 8a). In addition, some sectors of the Israeli society have called for a ban of trawling altogether. Therefore, we ran a scenario that applies the new fishing regulations with trawling eliminated within the first 3 years of the simulation (Fig. 8b) (Scn3). In addition, to quantify only the effects of these new fishing regulations, we ran a scenario keeping the biomass of alien species and temperature constant from their 2010 levels to 2060 (Scn4).

To predict the impact of sea warming on the ICS ecosystem, future SST projections of the study area were obtained from the Royal Netherlands Meteorological Institute Climate explorer (http://climexp.knmi.nl). We calculated SST projections under the four scenarios of greenhouse emissions (RCP2.6, RCP4.5, RCP6 and RCP8.5). As SST from this explorer did not match the SST from COPERNICUS, we calculated SST anomalies for the 2010–2060 period and these SST anomalies were applied to the COPERNICUS time series (Fig. 8c). Due to similar trends of the intermediate scenarios (RCP4.5 and RCP6), we applied only the RCP4.5 scenario. Therefore, the scenarios conducted to simulate potential impacts of sea warming were RCP2.6 (Scn5), RCP4.5 (Scn6) and RCP8.5 (Scn7). In these scenarios, fishing effort was kept constant from its 2010 levels to 2060.

To forecast future impacts of alien species, we forced the biomass of alien groups to follow current trends (Figure S3), while keeping fishing effort and SST constant from their 2010 levels to 2060 (Scn8).

In addition, we evaluated the combined impacts of the stressors simultaneously through three scenarios. In Scn9 (combination without forcing alien species), we merged scenarios 2 and 6, thus combining the new fishing regulations with an intermediate increase in SST, and we left alien species to change through the time (we did not force their biomass). In Scn10 (combination with forcing alien species), we merged scenarios 2, 6 and 8, thus combining the new fishing regulations, the intermediate increase in SST and an increase in the biomass of alien species following current trends. In Scn11 (combination with forcing alien species constant), we merged scenarios 2 and 6, thus combining the new fishing regulations, the intermediate increase in SST, and we force alien species to keep them at 2010 levels.

### Analysis

We analysed changes in the biomass of selected functional groups. These groups were chosen taking into account their inclusion in the time series fitting (see Corrales, *et al*.^46^) and considering their importance (economic and ecological importance, such as commercial species and vulnerable species). In addition, functional groups were aggregated taking into account their ecological role, taxonomy, habitat and between alien and native functional groups. Therefore, we defined separate groups as primary producers, zooplanktonic species, invertebrates, fishes and vulnerable species (which included sea turtles, sea birds and dolphins). Invertebrates and fishes were split into native and alien groups, and fishes were also divided between demersal and pelagic.

In addition, a selection of ecological indicators was used to evaluate the impacts of ecological changes on the ecosystem over time:

1. Total biomass (excluding detritus) (t·km^−2^), which included biomass of all the functional groups excluding detritus (detritus and discards). This indicator was used to quantify changes at the whole ecosystem level^20^.
2. Forage fish biomass (t·km^−2^), which included the biomass of benthopelagic fishes, small pelagic fishes, mackerel and horse mackerel. This indicator was analysed to quantify changes in the pelagic compartment^111^.
3. Invertebrate biomass (t·km^−2^), which included biomass of benthic invertebrate groups. This indicator was used to assess the dynamics of benthic invertebrates in the ecosystem, which tends to benefit from reductions in fish and predator biomass^112^.
4. Predatory biomass (t·km^−2^), which included biomass of all the groups with TL ≥4 and tends to decrease with increasing fishing impact in marine ecosystems^113^.
5. Kempton’s index, which expresses biomass diversity by considering those organisms with trophic levels ≥3 and tends to decrease with ecosystem degradation^114^.
6. Total catch (t·km^−2^·year^−1^), which includes the annual catches of the different fleets and provides an idea of total fisheries removals^111^.
7. Mean Trophic Level of the catch (mTLc), which expresses the TL of the catch, reflects the fishing strategy of the fleet and is used to quantify the impact of fishing^112^.
8. Mean Trophic Level of the community (mTLco), which expresses the Trophic Level (TL) of the whole ecosystem, reflects the structure of the ecosystem and is used to quantify the impact of fishing^113^.
9. Total System Throughput (t·km^−2^·year^−1^) (TST), which estimates the total flows in the ecosystem and is a measure of ecosystem size^115^.
10. Finn’s Cycling Index (FCI, %), which represents the proportion of the TST that is recycled in the system and is an indicator of stress and structural differences^116^.
11. Path length (PL), defined as the average number of compartments through which a unit of inflow passes, which is an indicator of stress^117^.

### Assessing uncertainty

Monte Carlo simulations and the Ecosampler plug-in were used to evaluate the impact of uncertainty in Ecopath input parameters (biomass, production and consumption rates) on Ecosim outputs (biomass and catch trends, and ecological indicators)^107,118,119^. We ran 500 Monte Carlo simulations for each scenario based on input parameter pedigree, which documents the quality of the input data (see Table S3 for confidence intervals of all input parameter), to determine the 5% and 95% confidence intervals for Ecosim outputs. Finally, a Spearman’s rank correlation test implemented in R software v 3.4.2 was used to assess the correlation between model outputs (predicted results without uncertainty analysis) with time.

## Electronic supplementary material

Supplementary information
