# Supplementary material for: Future scenarios of marine resources and ecosystem conditions in the Eastern Mediterranean under the impacts of fishing, alien species and sea warming
Source: Sci Rep. 2018 Sep 24;8:14284. doi: 10.1038/s41598-018-32666-x (PMC6155163; doi:10.1038/s41598-018-32666-x)
Supplement: Supplementary file 1 — Supplementary information [file 41598_2018_32666_MOESM1_ESM.docx]

**Future scenarios of marine resources and ecosystem conditions in the Eastern Mediterranean under the impacts of fishing, alien species and sea warming**

Corrales, X.^1,2*^, Coll, M.^2,3^, Ofir, E.^1^, Heymans, J.J.^4,5^, Steenbeek, J.^2,3^, Goren, M.^6^, Edelist, D.^7^, Gal, G^1^.

**Supplementary information**


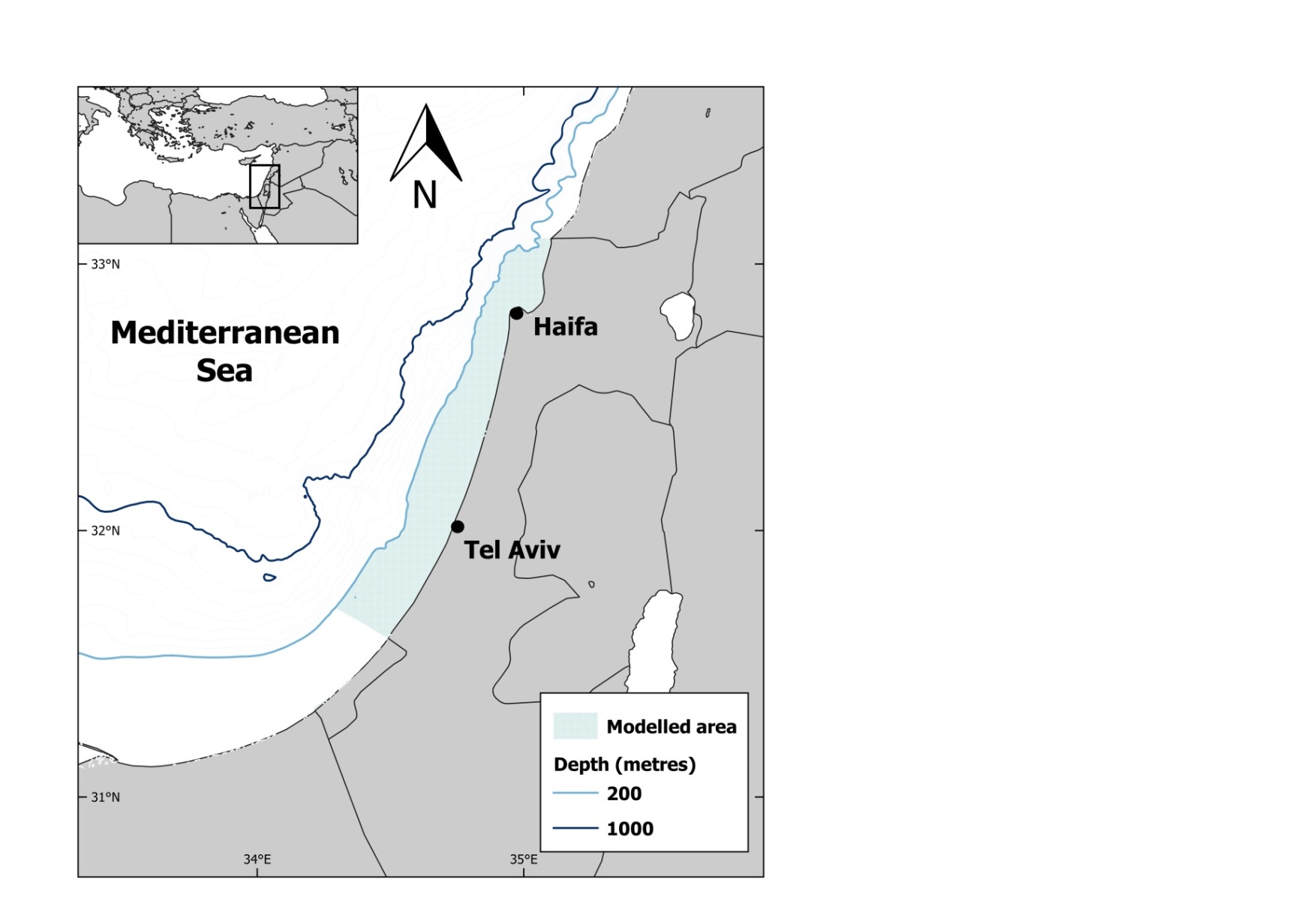


**Figure S1.** The study area encompassing the Israeli Mediterranean continental shelf (ICS) ecosystem and selected depth contours. Modelled area includes depths from 0 to 200 m.

**Figure S2.** a) Flow diagram of the Israeli Mediterranean continental shelf (ICS) model where functional groups represented by circles are organised by trophic level (y-axis) and habitat (x-axis). The size of the circles is proportional to the biomass of the functional groups and the thickness of the connecting lines is proportional to the magnitude of their trophic flows. The numbers identify the functional groups of the model (listed in Fig. 2b and Table A.1) and black circles represent alien groups. b) Mixed Trophic Impact (MTI) analysis of the Israeli Mediterranean continental shelf (ICS) model, which shows the direct and indirect impact in the food web that a hypothetical increase in the biomass of one functional group (impacting group) would have on the biomass of all other functional groups (impacted group). Negative (red) and positive (blue) impacts are represented.

**Figure S3.** Time series of forcing biomass of alien groups that were used in Scenario 8 and 10.

**Table S1.** Comparative ratios of biomass changes for the functional groups of the ICS ecosystem model between 2010 and 2060 (simulated period) under different scenarios.

| **Functional group** | | **Sc. 1** | **Sc. 2** | **Sc. 3** | **Sc. 4** | **Sc. 5** | **Sc. 6** | **Sc. 7** | **Sc. 8** | **Sc. 9** | **Sc. 10** | **Sc. 11** |
| --- | --- | --- | --- | --- | --- | --- | --- | --- | --- | --- | --- | --- |
| **1** | **Phytoplankton** | 1.01 | 1.04 | 1.08 | 0.99 | 1.00 | 1.01 | 1.01 | 1.04 | 1.02 | 1.05 | 0.98 |
| **2** | **Benthic primary producers** | 1.03 | 1.07 | 1.13 | 1.01 | 1.08 | 1.13 | 1.19 | 1.11 | 1.17 | 1.18 | 1.09 |
| **3** | **Micro and Mesozooplankton** | 0.97 | 0.90 | 0.80 | 1.02 | 1.00 | 0.98 | 0.98 | 0.90 | 0.95 | 0.88 | 1.04 |
| **4** | **Macrozooplankton** | 1.08 | 1.23 | 1.49 | 0.93 | 1.05 | 1.10 | 1.14 | 1.25 | 1.18 | 1.34 | 0.96 |
| **5** | **Gelatinous plankton** | 0.97 | 0.88 | 0.78 | 0.98 | 1.00 | 1.01 | 1.01 | 0.95 | 0.95 | 0.90 | 1.00 |
| **6** | **Polychaetes** | 1.04 | 1.14 | 1.25 | 0.98 | 1.02 | 1.03 | 1.04 | 0.81 | 1.12 | 0.85 | 1.08 |
| **7** | **Suprabenthos** | 1.00 | 1.04 | 1.06 | 1.00 | 0.99 | 1.00 | 1.00 | 0.89 | 1.04 | 0.89 | 1.03 |
| **8** | **Native Shrimps** | 1.03 | 1.05 | 1.05 | 0.94 | 1.07 | 1.11 | 1.13 | 0.81 | 1.18 | 0.80 | 1.09 |
| **9** | **Alien Shrimps** | 0.92 | 0.51 | 0.04 | 1.00 | 1.46 | 1.77 | 1.95 | 2.93 | 1.72 | 2.93 | 1.00 |
| **10** | **Native Crabs** | 0.97 | 0.89 | 0.80 | 0.84 | 1.05 | 1.08 | 1.11 | 0.85 | 1.07 | 0.82 | 1.03 |
| **11** | **Alien Crabs** | 0.47 | 0.52 | 0.10 | 1.00 | 0.86 | 0.94 | 1.06 | 4.30 | 1.57 | 4.30 | 1.00 |
| **12** | **Benthic invertebrates** | 1.03 | 1.10 | 1.16 | 1.03 | 1.01 | 1.02 | 1.03 | 0.91 | 1.08 | 0.91 | 1.06 |
| **13** | **Benthic cephalopods** | 1.01 | 0.35 | 0.00 | 0.17 | 1.30 | 1.65 | 1.82 | 0.02 | 1.15 | 0.00 | 0.93 |
| **14** | **Benthopelagic cephalopods** | 0.88 | 0.57 | 0.25 | 0.67 | 0.93 | 0.97 | 1.00 | 0.08 | 0.69 | 0.02 | 0.96 |
| **15** | **Mullets** | 3.90 | 9.56 | 21.43 | 8.66 | 0.04 | 0.00 | 0.00 | 0.96 | 0.75 | 0.01 | 0.62 |
| **16** | **Goatfishes** | 0.99 | 1.26 | 1.65 | 1.00 | 1.06 | 1.11 | 1.15 | 3.56 | 1.46 | 3.56 | 1.00 |
| **17** | **Hake** | 1.06 | 3.08 | 6.95 | 3.95 | 0.21 | 0.08 | 0.01 | 2.74 | 1.33 | 3.32 | 1.52 |
| **18** | **Flatfishes** | 0.77 | 1.40 | 1.92 | 1.61 | 0.64 | 0.60 | 0.57 | 0.21 | 1.15 | 0.40 | 1.53 |
| **19** | **Rocky fishes** | 1.02 | 0.95 | 0.91 | 0.98 | 0.68 | 0.55 | 0.05 | 0.60 | 0.48 | 0.03 | 0.52 |
| **20** | **Small native dem. fish** | 0.65 | 0.83 | 0.63 | 0.87 | 0.50 | 0.16 | 0.00 | 0.00 | 0.26 | 0.00 | 0.59 |
| **21** | **Large native dem. fish** | 0.31 | 3.50 | 5.80 | 3.80 | 0.50 | 0.70 | 1.05 | 7.20 | 4.69 | 12.23 | 3.51 |
| **22** | **Alien herbivores** | 1.05 | 0.50 | 0.26 | 1.00 | 1.11 | 1.25 | 1.31 | 2.42 | 0.67 | 2.42 | 1.00 |
| **23** | **Earlier alien dem. fish** | 1.27 | 0.13 | 0.00 | 1.00 | 1.36 | 1.54 | 1.63 | 2.85 | 0.19 | 2.85 | 1.00 |
| **24** | **New alien dem. fish** | 1.73 | 1.90 | 2.56 | 1.00 | 1.74 | 1.87 | 1.94 | 4.71 | 1.99 | 4.71 | 1.00 |
| **25** | **Alien lizardfish** | 1.65 | 2.77 | 5.24 | 1.00 | 1.90 | 1.96 | 1.91 | 4.85 | 3.10 | 4.85 | 1.00 |
| **26** | **Demersal fishes (upper slope)** | 0.97 | 0.93 | 0.90 | 0.77 | 1.13 | 1.18 | 1.23 | 0.36 | 1.27 | 0.60 | 1.29 |
| **27** | **Benthopelagic fishes** | 0.93 | 0.88 | 0.81 | 1.02 | 0.92 | 0.91 | 0.91 | 0.79 | 0.87 | 0.76 | 1.00 |
| **28** | **Mesopelagic fishes** | 1.15 | 1.25 | 1.39 | 1.13 | 1.13 | 1.11 | 1.09 | 1.82 | 1.18 | 1.74 | 0.98 |
| **29** | **Demersal sharks** | 1.07 | 1.36 | 1.96 | 1.28 | 1.09 | 1.12 | 1.14 | 1.56 | 1.42 | 1.95 | 1.27 |
| **30** | **Rays and Skates** | 1.02 | 1.40 | 2.15 | 1.41 | 1.00 | 1.01 | 1.01 | 1.25 | 1.36 | 1.62 | 1.32 |
| **31** | **Small pelagic fishes** | 0.97 | 0.96 | 0.91 | 1.14 | 0.96 | 0.93 | 0.91 | 1.16 | 0.95 | 1.11 | 1.01 |
| **32** | **Horse mackerel** | 0.80 | 0.82 | 0.72 | 1.02 | 0.50 | 0.24 | 0.00 | 0.56 | 0.27 | 0.13 | 0.5 |
| **33** | **Mackerel** | 1.32 | 1.36 | 1.47 | 1.54 | 1.56 | 1.76 | 2.02 | 0.65 | 1.66 | 1.30 | 2.17 |
| **34** | **Native medium pelagic fishes** | 0.94 | 0.92 | 0.83 | 1.08 | 0.94 | 0.91 | 0.90 | 0.89 | 0.91 | 0.93 | 1.09 |
| **35** | **Alien medium pelagic fishes** | 2.14 | 2.44 | 3.27 | 1.00 | 2.27 | 2.17 | 2.06 | 5.33 | 2.49 | 5.33 | 1.00 |
| **36** | **Large pelagic fishes** | 0.99 | 1.10 | 1.07 | 1.19 | 0.99 | 0.97 | 0.96 | 1.00 | 1.08 | 1.15 | 1.17 |
| **37** | **Turtles** | 0.65 | 1.54 | 1.84 | 1.56 | 0.65 | 0.64 | 0.62 | 0.74 | 1.64 | 1.68 | 1.62 |
| **38** | **Sea birds** | 0.95 | 0.78 | 0.48 | 0.87 | 0.89 | 0.82 | 0.78 | 0.97 | 0.71 | 0.75 | 0.77 |
| **39** | **Dolphins** | 0.92 | 0.89 | 0.92 | 1.00 | 0.90 | 0.89 | 0.89 | 0.84 | 0.86 | 0.85 | 0.98 |
| **40** | **Detritus** | 1.01 | 1.03 | 1.06 | 0.99 | 1.00 | 1.00 | 1.01 | 1.04 | 1.01 | 1.04 | 0.99 |
| **41** | **Discards** | 0.95 | 0.61 | 0.04 | 0.59 | 0.81 | 0.70 | 0.62 | 0.83 | 0.45 | 0.42 | 0.49 |

**Table S2.** Temperature response functions included in the Israeli Mediterranean continental shelf ecosystem model.

|  |  |  | | | |
| --- | --- | --- | --- | --- | --- |
|  | **Functional group** | **Minimum** | **10th quantile** | **90th quantile** | **Maximum** |
| 8 | Native Shrimps | 8 | 17 | 26 | 30 |
| 9 | Alien Shrimps | 11.8 | 18 | 27 | 32 |
| 10 | Native Crabs | 7.8 | 18.6 | 27.7 | 30 |
| 11 | Alien Crabs | 13.5 | 19 | 28.4 | 32 |
| 15 | Mullets | 5 | 12.3 | 20 | 26.5 |
| 16 | Goatfishes | 12 | 17.5 | 26 | 31 |
| 17 | Hake | 5 | 11.1 | 19.5 | 28 |
| 19 | Rocky fishes | 8.8 | 10.8 | 20.5 | 27 |
| 20 | Small Native dem. fish | 9.9 | 14.9 | 20.5 | 27.5 |
| 21 | Large Native dem. fish | 10.3 | 17.1 | 24 | 28.1 |
| 22 | Alien herbivorous | 16 | 17.5 | 25 | 30 |
| 23 | Earlier alien dem. fish | 13 | 15 | 26 | 31 |
| 24 | New alien dem. fish | 13.5 | 17 | 27 | 32 |
| 25 | Alien lizardfish | 14 | 18 | 25 | 29 |
| 27 | Benthopelagic fishes | 5 | 14.5 | 23.5 | 29.1 |
| 29 | Demersal sharks | 11.8 | 15.1 | 27.7 | 31.9 |
| 30 | Rays and Skates | 10.4 | 17.2 | 27.8 | 32 |
| 31 | Small pelagic fishes | 9.1 | 16.3 | 26.6 | 31 |
| 32 | Horse mackerel | 10.9 | 14.1 | 20 | 26 |
| 33 | Mackerel | 15 | 17 | 24 | 30 |
| 34 | Native medium pelagic fishes | 13 | 15 | 23 | 30 |
| 35 | Alien medium pelagic fishes | 14 | 17 | 25 | 32 |
| 36 | Large pelagic fishes | 9.4 | 17 | 27 | 31.5 |

**Table S3.** Confidence intervals (expressed as % of variation) for each input parameters in the Ecopath model used to describe the uncertainty.

| **Functional group** | | **Biomass** | **P/B** | **Q/B** |
| --- | --- | --- | --- | --- |
| **1** | **Phytoplankton** | 10 | 10 | - |
| **2** | **Benthic primary producers** | 30 | 20 | - |
| **3** | **Micro and Mesozooplankton** | 30 | 30 | 30 |
| **4** | **Macrozooplankton** | 30 | 30 | 30 |
| **5** | **Gelatinous plankton** | 30 | 30 | 30 |
| **6** | **Polychaetes** | 30 | 30 | 30 |
| **7** | **Suprabenthos** | 30 | 30 | 30 |
| **8** | **Native Shrimps** | 30 | 20 | 20 |
| **9** | **Alien Shrimps** | 30 | 20 | 20 |
| **10** | **Native Crabs** | 30 | 20 | 20 |
| **11** | **Alien Crabs** | 30 | 20 | 20 |
| **12** | **Benthic invertebrates** | 30 | 30 | 30 |
| **13** | **Benthic cephalopods** | 30 | 30 | 30 |
| **14** | **Benthopelagic cephalopods** | 30 | 30 | 30 |
| **15** | **Mullets** | 15 | 20 | 20 |
| **16** | **Goatfishes** | 15 | 20 | 20 |
| **17** | **Hake** | 15 | 20 | 20 |
| **18** | **Flatfishes** | 15 | 20 | 20 |
| **19** | **Rocky fishes** | 15 | 20 | 20 |
| **20** | **Small native dem. fish** | 15 | 20 | 20 |
| **21** | **Large native dem. fish** | 15 | 20 | 20 |
| **22** | **Alien herbivores** | 30 | 20 | 20 |
| **23** | **Earlier alien dem. fish** | 15 | 20 | 20 |
| **24** | **New alien dem. fish** | 15 | 20 | 20 |
| **25** | **Alien lizardfish** | 15 | 20 | 20 |
| **26** | **Demersal fishes (upper slope)** | 15 | 20 | 20 |
| **27** | **Benthopelagic fishes** | 30 | 20 | 20 |
| **28** | **Mesopelagic fishes** | 30 | 20 | 20 |
| **29** | **Demersal sharks** | 15 | 20 | 20 |
| **30** | **Rays and Skates** | 15 | 20 | 20 |
| **31** | **Small pelagic fishes** | 30 | 20 | 20 |
| **32** | **Horse mackerel** | 30 | 20 | 20 |
| **33** | **Mackerel** | 30 | 20 | 20 |
| **34** | **Native medium pelagic fishes** | 30 | 20 | 20 |
| **35** | **Alien medium pelagic fishes** | 20 | 20 | 20 |
| **36** | **Large pelagic fishes** | 20 | 20 | 20 |
| **37** | **Turtles** | 30 | 30 | 30 |
| **38** | **Sea birds** | 15 | 30 | 20 |
| **39** | **Dolphins** | 15 | 30 | 20 |
